# Establishment of a Real-Time Recombinase Polymerase Amplification Assay for the Detection of Avian Reovirus

**DOI:** 10.3389/fvets.2020.551350

**Published:** 2020-09-22

**Authors:** Lei Ma, Hongfei Shi, Mingliang Zhang, Yuwei Song, Kunpeng Zhang, Feng Cong

**Affiliations:** ^1^School of Biotechnology and Food Engineering, Anyang Institute of Technology, Anyang, China; ^2^Henan Provincial Engineering Laboratory of Insects Bio-reactor, China-UK-NYNU-RRes Joint Libratory of Insect Biology, Nanyang Normal University, Nanyang, China; ^3^Guangdong Key Laboratory of Laboratory Animals, Guangdong Laboratory Animals Monitoring Institute, Guangzhou, China; ^4^Henan Joint International Research Laboratory of Veterinary Biologics Research and Application, Academician Workstation of Animal Disease Control and Nutrition Immunity in Henan Province, Anyang, China

**Keywords:** avian reovirus, recombinase polymerase amplification, rapid detection, specific, sensitive

## Abstract

Avian reovirus (ARV) infection results in multiple disease manifestations in chicken. A rapid detection method will contribute to early diagnosis and control of the virus infection. The recombinase polymerase amplification (RPA) technology is a nucleic acid amplification method which is experiencing rapid development. In present study, a real-time reverse transcription (RT)-RPA assay was developed for the detection of ARV. The limit of detection of the real-time RT-RPA was 10^2^ copies/μL of ARV genomic RNA standard in 95% of cases. The RT-RPA assay also exhibited remarkable specificity. When the nucleic acids of CRV and other common avian pathogens were subjected to the RT-RPA test, only ARV tested positive, all the other pathogens tested negative. Furthermore, the practicality of the RT-RPA assay in field was confirmed by testing 86 clinical samples. The clinical samples were also detected by qRT-PCR. The detection result by RT-RPA was 96.5% agreement with that of qRT-PCR. As a result of the simplicity and convenience of the assay with high sensitivity and specificity, the probe-based RT-RPA will be an alternative diagnostic assay for the detection of ARV in resource-limited settings.

## Introduction

Avian reoviruses (ARVs) play significant roles in multiple disease manifestations in birds (1). ARV could infect various kinds of birds including chickens, turkeys, goslings, ducks and partridge, and leads to many kinds of disease syndromes including arthritis/tenosynovitis, intestinal diseases, respiratory diseases, diseases of the central nervous system (2–7). Since its first report in 1960s, ARV has been detected and isolated from domestic and wild birds across the world (6–15). Co-infections of ARV with other pathogens in commercial broiler chickens in intensive breeding farms lead to low feed conversion, poor weight gains and reduced marketability of affected chickens, resulting into huge economic losses. To enhance monitoring and diagnosis for avian reovirus (ARV) infection, a simple, sensitive and effective detection method would be essential for the effective control and prevention of ARV in poultry flocks.

Virus isolation is a classical detection method for the diagnosis of ARV in clinical samples (1). The virus culture in monolayer cells is time-consuming which takes a period of 5–7 days to develop viral cytopathic effects (CPE) (10). Furthermore, 2-3 passages are given to allow virus to grow as ARV are slow growing. Hence it is standard method to give 2-3 passages before reporting as negative (10). In comparison to culture method, molecular techniques are more sensitive and rapid, but it need sophisticated thermal cycling equipment and elaborate protocols (16–18). Thus, a user-friendly and roust diagnostic assay for the detection of ARV nucleic acids in clinical samples will make prevention and control program more effective. The isothermal amplification technique, which is carried out at an optimal single temperature, is faster and more convenient than PCR approach. Among the isothermal nucleic acid amplification methods, the loop-mediated isothermal amplification assay (LAMP) was commonly used (5), it still took about an hour to obtain the result. Although RPA method was a latterly invented technique, it has attracted much attention for the last few years (19). RPA reaction was initiated by three proteins, including a recombinase, a strand-displacing DNA polymerase and a single-stranded DNA-binding protein at a single optimum temperature between 37 and 42°C. Accumulating studies demonstrates that RPA technique has been successfully used in rapid detection of bacteria, viruses, and parasites (20–24). Endpoint detection of RPA products could be carried out by nucleic acid electrophoresis and lateral flow dipstick (LFD) (25). Compared to electrophoresis and LFD, a probe-based RPA assay can generate real-time amplification result in <20 min without an additional process after amplification (21).

In this study, we aim to establish a probe-based RT-RPA and evaluate its performance for the detection of ARV in field samples.

## Materials and Methods

### Viruses

The virus strains infectious bursal disease virus (IBDV) B87, reticuloendotheliosis virus (REV) C15 and the vaccine strain Marek's disease virus (MDV) 814 were purchased from Guangdong Animal Epidemic Prevention and Material Reserve Center. Chicken anemia virus (CAV) GD-2014, newcastle disease virus (NDV), ARV S1133, Avian influenza virus (AIV) H7N2 subtype, infectious bronchitis virus (IBV), infectious laryngotracheitis virus (ILTV), Mycoplasma gallisepticum (MG) and Mycoplasma synoviae (MS) were preserved in our laboratories.

### Generation of RNA Molecule Standard

The target gene of the RT-RPA assay was the S1 fragment. S1 segment was amplified for the preparation of the RNA standard. A specified primer set (forward primer: 5′-ACTGTCATTGACTTCGAACG-3′, reverse primer: 5′-CTCGAGTACACCCCATACGC-3′) were designed to amplify the S1 conserved nucleotide sequence. Total nucleic acids of the cell suspension infected with ARV S1133 were isolated for RT-PCR reaction. The RT-PCR product was subjected to electrophoresis on a 2% agrose gel and purified. The purified amplicons were inserted into the pGEM-T vector (Promega, USA) and *in vitro* transcribed as the method previously reported (20). The transcribed RNA concentration was determined using Nano2000 (GE, USA). The RNA copy numbers were calculated as the method previously reported (26).

### Primers and Probe Design

To establish the RT-RPA assay, the primer and probe were designed based on the nucleotide sequence of ARV S1133 (Genbank ID: MG822668.1) following the instruction manual provided by TwistDx Inc. Cambridge, UK, and synthesized by BGI Genomics (Shenzhen, China). The nucleotide sequences of the primers and probe were summarized in Table 1.

**Table 1:** RPA primers and probe.

**Primer name**	**Primer sequence (5^**′**^to3^**′**^)**	**Position**	**Amplicon size (bp)**
ARV-1F	CTGTTCTCAACGAGTTTCTTTAACATCATA	543–572	209
ARV-1R	TTAAATCGAAGGTTAATAACACGAC	751–727	
ARV-2F	TTCTCAACGAGTTTCTTTAACATCATACT	546–574	184
ARV-2R	ATGTCACTTAAATCGAAGGTTAATAACACG	758–729	
ARV-3F	CTTCTGTTCTCAACGAGTTTCTTTAACATC	540–569	135
ARV-3R	CTTCCATGACAGTGAGCGTTAACGGTCATG	674–645	
ARV-4F	CAACGAGTTTCTTTAACATCATACTCGGCG	550–579	138
ARV-4R	ATAATCAGTGCGTCTTCCATGACAGTGAGC	687–658	
probe	ACTAATGCAATTTCGGTGGATGGCACGGGG (dT-FAM)(THF)C(dT-BHQ1)AACGGATCATCTGAT(C3-spacer)	588–636	

### RT-RPA Procedure

The RT-RPA reaction was performed by TwistAmp® RT exo kit (TwistDx Inc. Cambridge, UK). The protocol for preparation of the RT-RPA assay was following the manufacturer's instructions. Real-time RPA reaction was performed in a 50 μL volume. In a reaction tube containing the dried enzyme pellets, 29.5 μL of rehydration buffer, 1 μL template, 2.1 μL of each primer (10 μm), 0.6 μL probe, 2.5 μL magnesium acetate and 12.2 μL water were added. The reaction tubes were inverted thoroughly and centrifuged briefly. Then the tubes were immediately inserted into the fluorescence detector Deaou-308C (DEAOU Biotechnology, Guangzhou, China) at 39°C. The tubes were firstly incubated for 4 min, and mixed thoroughly. The tubes were then incubated for 16 min at 39°C. The fluorescence was measured for 40 times at 30s internal. If a sample generated an amplification curve above the threshold of the negative control, it was considered positive and below threshold as negative.

### Sensitivity and Specificity of the Assay

The minimal detection limit of the RT-RPA assay was analyzed by the 10-fold serially diluted RNA standards ranged from 10^0^ copy/μL-10^6^ copies/μL. Each RNA standard dilution was tested by the RT-RPA assay in 8 replicates. The cross-reaction of the assay also evaluated. The pathogens used for specificity test included IBDV, REV, MDV, CAV, AIV, NDV, IBV, ILTV, MG, and MS. ARV S1133 was used as positive control.

### Clinical Samples

To evaluate the practicality of the developed real-time RT-RPA assay, 86 tendon tissue specimens were collected from the chickens suspected to be infected with ARV in a poultry farm in Guangdong province. All the animal procedures were approved by the Office of Laboratory Animal.

Management of Guangdong Laboratory Animals Monitoring Institute, Guangzhou, China. One μg tendon was placed in 1.5-mL Eppendorf tube. The tendon was cut by scissors and prepared as tissue homogenate with the disposable pestle (Axygen Inc., USA) by adding 250 μL phosphate buffered saline (PBS). The tendon homogenate was frozen and thawed twice and clarified by low-speed centrifugation, and the supernatants were collected for the extraction of nucleic acids. Total nucleic acids of the specimens were extracted using a QIAamp Viral RNA Mini Kit (Qiagen, Germany) and tested by both real-time fluorescence RT-PCR and RT-RPA. The real-time fluorescence RT-PCR was performed in accord with a previously reported protocol (17). A minimum of 39 copies/μL of ARV genomic RNA could be detected by the assay. RNA volume and total reaction volume used for real-time RT-PCR were the same as that for RT-RPA.

## Results

### Screening of the Primer-Probe Set

**Figure 1:**
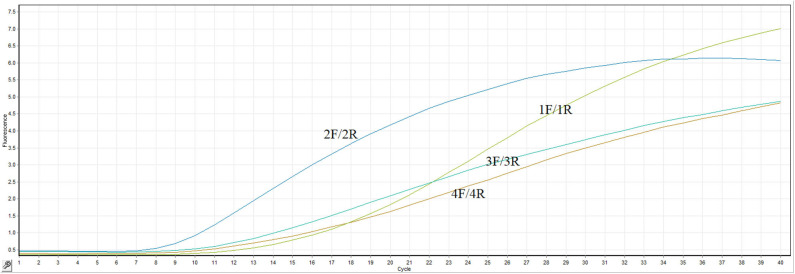
Primer sets screening. Four primers pairs were evaluated by the probe-based RT-RPA assay.

The most important thing for optimization of the RT-RPA assay is to screen an efficient primer-probe set. In present study, four primer combinations with one probe were evaluated by the real-time RT-RPA assay. As clearly illustrated in Figure 1, the primer pair 2F/2R took the least time to generate test result among all the primer combinations. The fluorescence signal of primers 2F/2R increased significantly from 4 min, whereas the other three primer pairs took about 5 min. Besides, the fluorescence amplitude of 2F/2R along with the time was consistently highest among all the primer pairs. Thus, the primers-probe set (2F/2R) was used to establish the RT-RPA assay for the detection of ARV.

### Specificity Test

**Figure 2:**
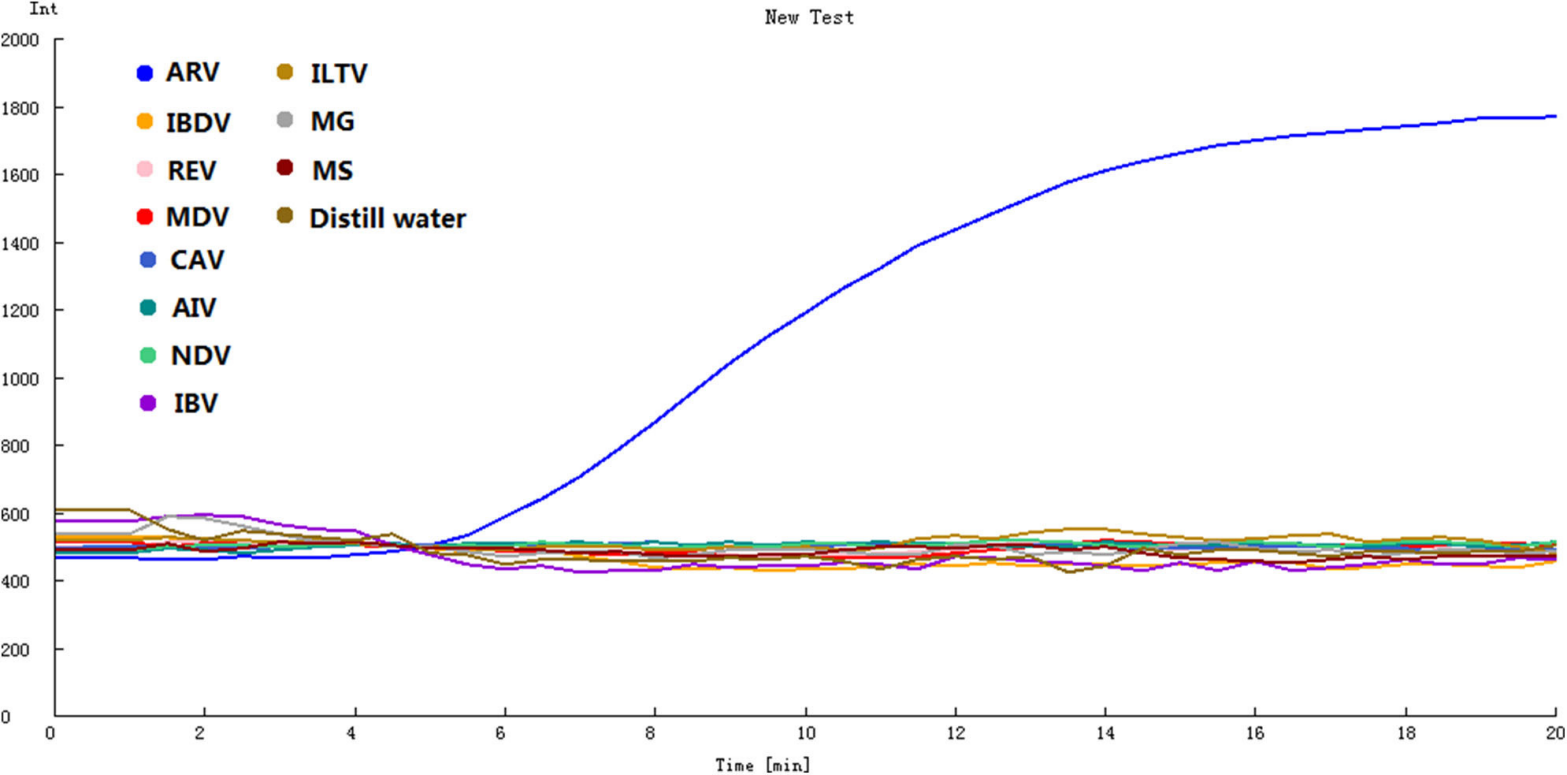
Specificity of the real-time RT-RPA assay. IBDV, REV, MDV, CAV, AIV, NDV, IBV, ILTV, MG, MS were tested by the assay. ARV was detected by the assay as the positive control, and distilled water was the negative control.

To determine the specificity of the assay, ARV and 10 other avian pathogens were detected by the assay. The remarkable fluorescence increase was only observed for ARV S1133 but not the other 10 avian pathogens or the negative control sample (Figure 2), indicating that the RT-RPA assay using the optimal primers-probe set was ARV-specific and had no cross-reactions with other pathogens.

### Detection Limit of the Assay

**Figure 3:**
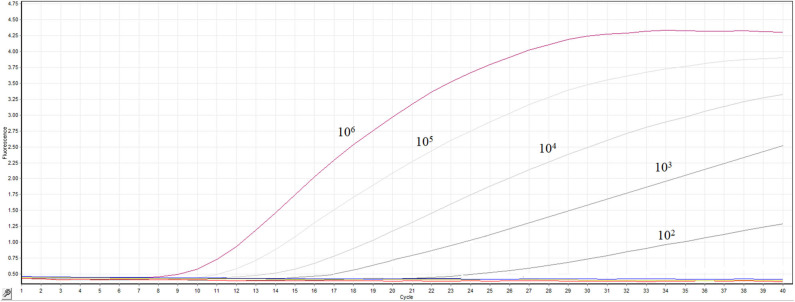
Sensitivity the real-time RT-RPA assay. A range of serially diluted RNA standards from 10^0^ copy/μL-10^6^ copies/μL were detected by the assay.

The transcribed RNA was 10-fold serially diluted (10^0^ copy/μL-10^6^ copies/μL) and detected by the real-time RT-RPA assay. The templates containing 10^6^ −10^2^ RNA molecules produced fluorescence curves, while other templates and the distilled water didn't produced fluorescence curve (Figure 3). The RNA dilutions were tested in 8 replicates by the RT-RPA assay to assess the repeatability of the assay (Supplementary Figures 1–6). The RNA dilutions ranging from 10^6^-10^2^ copies were all positive by RT-RPA in 8 replicates. However, only one test was positive when RNA concentration was 10 copies; and all the 8 replicates were negative when one copy per reaction was used. The exact detection limit of the assay was 10^2^ copies molecules in 95% of cases determined by the probit regression analysis.

To verify the sensitivity of the method, more strains of ARV were tested. Blast analysis (https://blast.ncbi.nlm.nih.gov/Blast.cgi) showed that the sequences of the primer-probe set share 100% similarity with most of the ARV strains. Meanwhile, several point mutations were present on some ARV strains (ARV strain GX110058, ARV strain B-98 et.al) at the binding site of the primer-probe sequence. So ARV strain GX110058 segment S1 (GenBank: KF741742.1) and strain B-98 segment S1 (GenBank: DQ643974.1) were synthesized by BGI Genomics (Shenzhen, China) and cloned in pGEM-T vector. The sensitivity of the RT-RPA assay was evaluated by the above method. The result showed that the assay could detect 10^2^ copies ARV strain GX110058 and ARV strain B-98 S1 molecules, respectively (Supplementary Figures 7, 8), confirming that several point mutations have little effect on the sensitivity of the RT-RPA assay which was reported previously (21).

### Performance of the RT-RPA Assay on Clinical Samples

**Figure 4:**
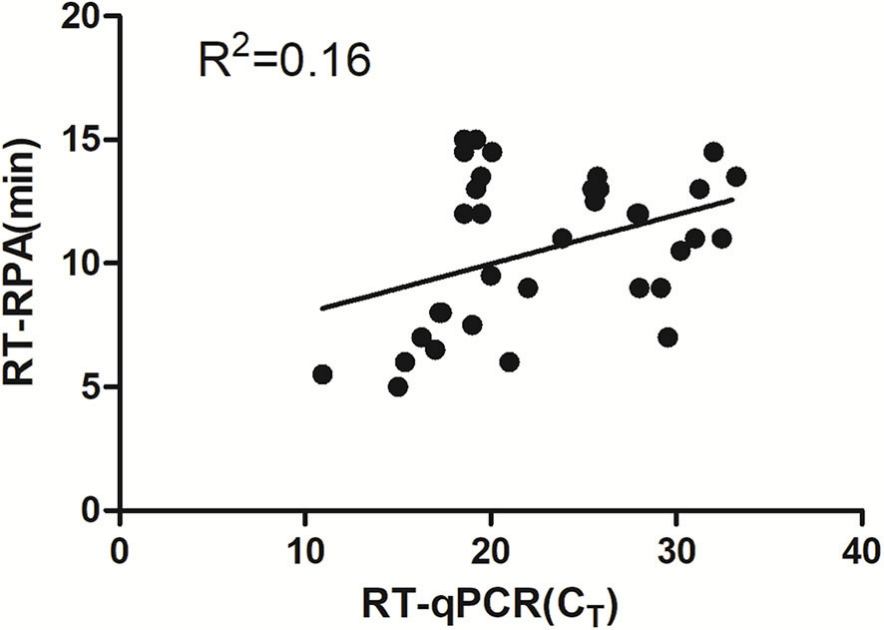
Validation of the RT-RPA assay on the clinical samples. RT-RPA threshold times (TT, y axis) and qRT-PCR cycle threshold values (CT, x axis) of the clinical samples were compared by Prism software. *R*^2^ value was 0.16.

Eighty-six tendon tissue specimens which were collected from the chickens in a poultry farm were detected by the assay developed above. Of these samples, 38 ARV-positive samples and 48 ARV-negative samples were confirmed by RT-RPA. All the samples were detected by a previously reported qRT-PCR assay. The qRT-PCR result showed that 41 samples were ARV-positive and 45 samples were ARV-negative. The detailed test results were displayed in the Table 2. There were 3 positive samples determined by qRT-PCR, but negative by RT-RPA. The Ct values of the three samples were >35, indicating low viral loads. The minimum detection limit of the qRT-PCR assay was 39 copies/μL of ARV genomic RNA. Ct value was 34.9 when the input copy number was 100 copies RNA (17). The Ct values >35 suggested ARV genomic RNA of the three samples were less than the detection limit of RT-RPA. The coincidence rate was 96.5% between the test results of RT-RPA and RT-qPCR (Table 2). The threshold time (TT) values and C_T_ values showed a poor correlation by linear regression analysis, suggesting the real-time RT-RPA assay could be used to qualitative detection of ARV (Figure 4). The poor correlation has been reported in several RPA studies (20, 21). Currently, there were few studies reporting the possible reasons. The possible explanation was that the extracts of the samples that contained the other irrelevant nucleic acids besides ARV may affect the efficiency of enzymes in the RPA reaction.

**Table 2:** Comparison between the results of RT-RPA and RT-qPCR.

			**RT-qPCR**		**CR**
		**Positive**	**Negative**	**Total**	
RT-RPA	Positive	38	0	38	96.5%
	Negative	3	45	48	
	Total	41	45	86	

CR: Coincidence rate. CR= (38+45)/86*100%.

## Discussion

In present study, we established a probed-based isothermal amplification assay for rapid and sensitive diagnosis of ARV. The fluorescence-based RT-RPA assay was carried out at a consistent temperature of 39°C, and the amplification took about 20 min to finish (Figure 1). Fluorescence signals could be real-time monitored by a portable device. Only nucleic acid from ARV could be amplified by the assay. The other viruses which are common infectious pathogens in chicken were not amplified by the assay, suggesting remarkable specificity of the RT-RPA assay (Figure 2). The real-time RT-RPA assay could detect as few as 100 genomic copies per reaction. The assay was applied to detect 86 clinical samples. The detection result of the clinical samples by RT-RPA was 96.5% consistent with that of the qRT-PCR method. Therefore, the RT-RPA assay could feasibly be used to rapid detection of ARV and had the potential to apply in field.

Primer design was of vital importance in the establishment of a RT-RPA assay with high sensitivity and specificity. Four primer pairs combined with a probe were screened for the optimal performance of the assay. The primer pair 2F/2R took the least time to generate test result among all the primer combinations. The fluorescence signal of primers 2F/2R increased significantly from 4 min, whereas the other three primer pairs took about 5 min. Besides, the fluorescence amplitude of 2F/2R along with the time was consistently highest among all the primer pairs. Thus, the optimal primer pair 2F/2R was used in the RT-RPA assay.

RPA which was invented in 2006 has been experiencing rapid development (19, 27). The RPA method was simpler and faster compared to other detection methods. For example, ARV diagnosis by cell culture took at least 48 h to obtain the result. Real-time qRT-PCR was faster, but still took about 2 h to obtain the ultimate result (17), expensive instrument and professional team were indispensable. In the LAMP assay for the detection of ARV, six primers were required (5). Hence, compared to the previously reported methods, the advantages of the RPA method are obvious. The real-time RT-RPA assay developed in present study only required two primers and one probe, the test result could be obtained in <20 min. As illustrated in Figure 1, the template containing 10^6^ copies RNA molecules took about 5 min to obtain the amplification curve in the RT-RPA assay. To our best knowledge, the RT-RPA assay is the most rapid among all the previously reported methods for the detection of ARV (17, 28). Low cost is another advantage of the RPA assay.

For rapid detection of ARV in field with limited resources, RPA method has huge advantages including its portability, simplicity, and short turnaround time. A RPA-based portable laboratory has been established for field detection of dengue virus (29). In our study, 86 specimens were simultaneously tested by the RT-RPA and qRT-PCR. The detection result of the RT-RPA assay was 96.5% in agreement with that of the qRT-PCR assay, indicating good performance of the assay and potential for detecting ARV in field. In summary, the highly sensitive RT-RPA is suitable for extensive applications in laboratories, which are specifically designed for ARV detection and surveillance.

## Data Availability Statement

The original contributions presented in the study are included in the article/Supplementary Material, further inquiries can be directed to the corresponding author/s.

## Ethics Statement

The animal study was reviewed and approved by Animal experiments were approved by the Office of Laboratory Animal Management of Guangdong Laboratory Animals Monitoring Institute, Guangzhou, China.

## Author Contributions

LM, HS, and FC designed the study and drafted the manuscript. LM and MZ conducted the experiments, data analysis, and participated in drafting of the manuscript. YS and KZ helped with the experiments. All the authors read and approved the final manuscript.

## Conflict of Interest

The authors declare that the research was conducted in the absence of any commercial or financial relationships that could be construed as a potential conflict of interest.
