# Supplementary material for: Establishment of a Real-Time Recombinase Polymerase Amplification Assay for the Detection of Avian Reovirus
Source: Front Vet Sci. 2020 Sep 22;7:551350. doi: 10.3389/fvets.2020.551350 (PMC7536300; doi:10.3389/fvets.2020.551350)
Supplement: Supplementary file 1 [file Table_1.DOCX]

Fig S1


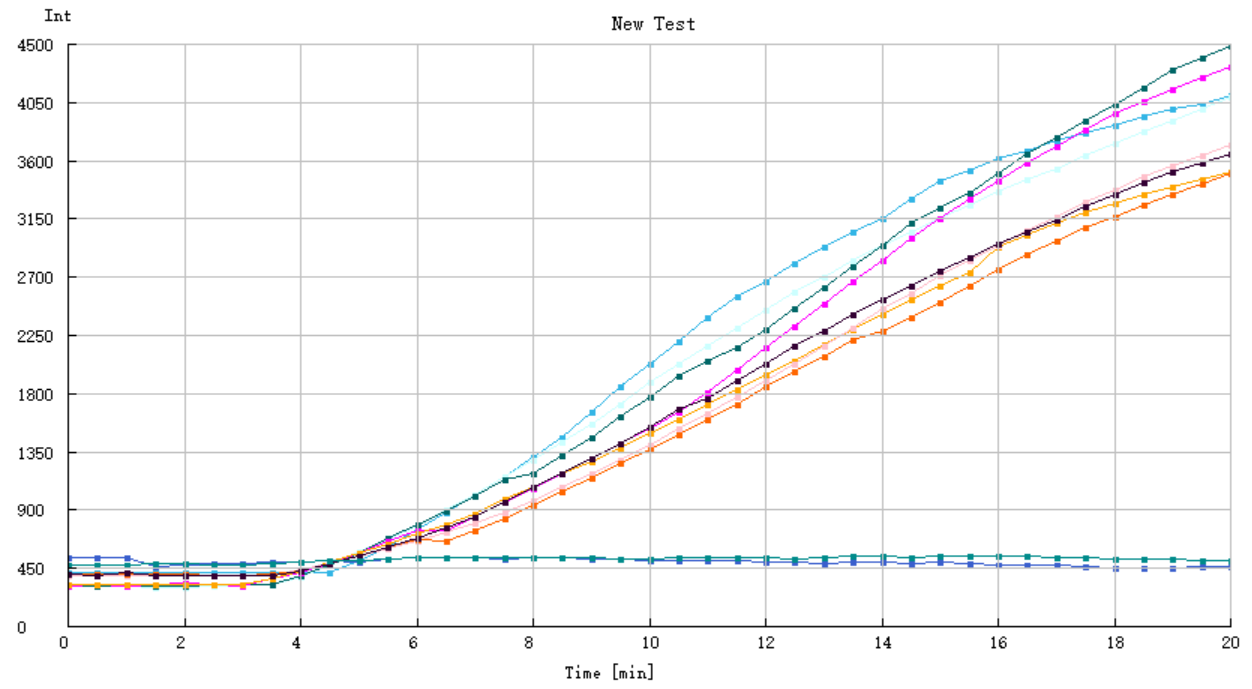


Fig S1 Eight replicates of RNA standard (10^6^ copies/μL) were detected by the assay.

Fig S2


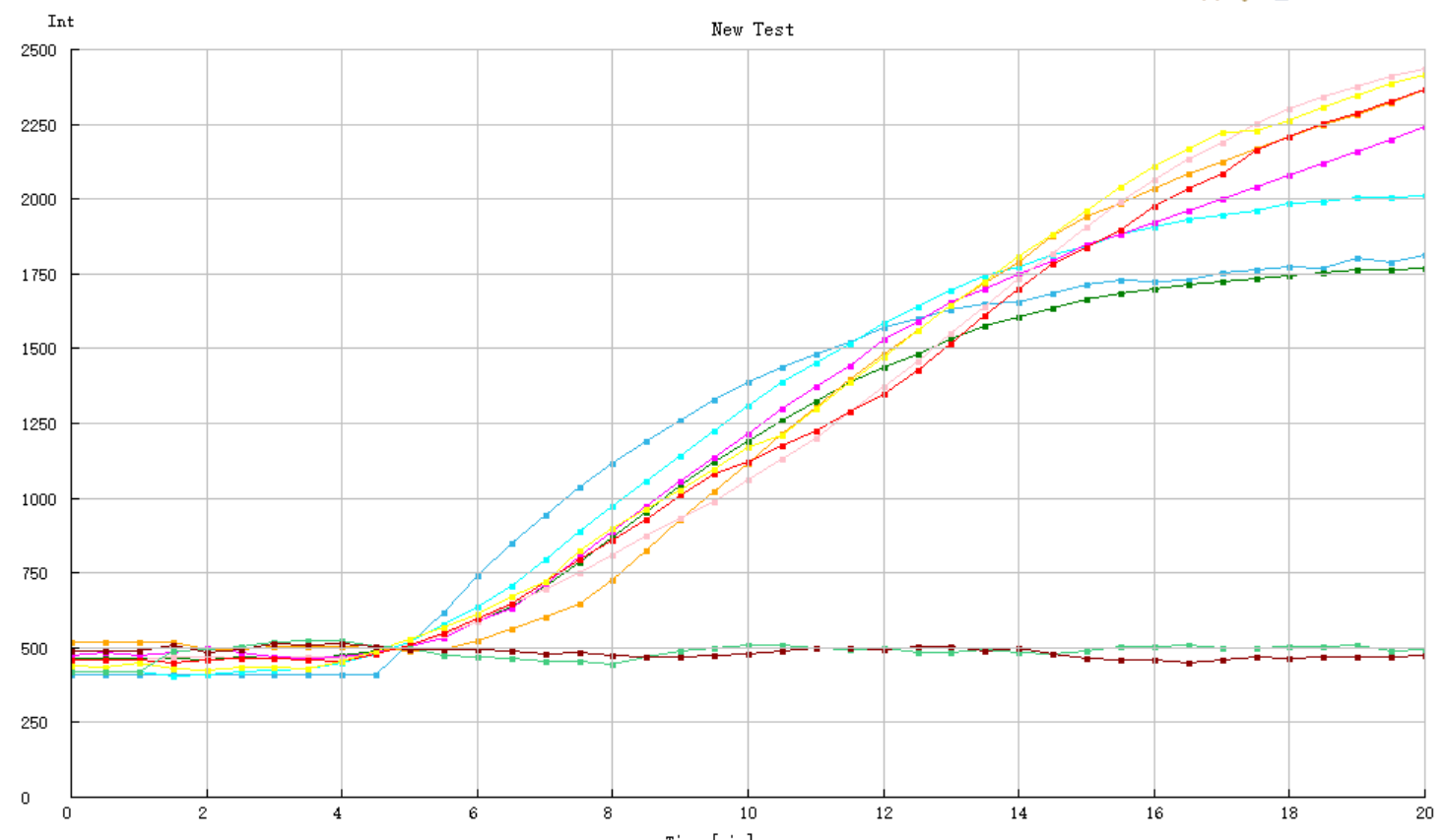


Fig S2 Eight replicates of RNA standard (10^5^ copies/μL) were detected by the assay.

Fig S3


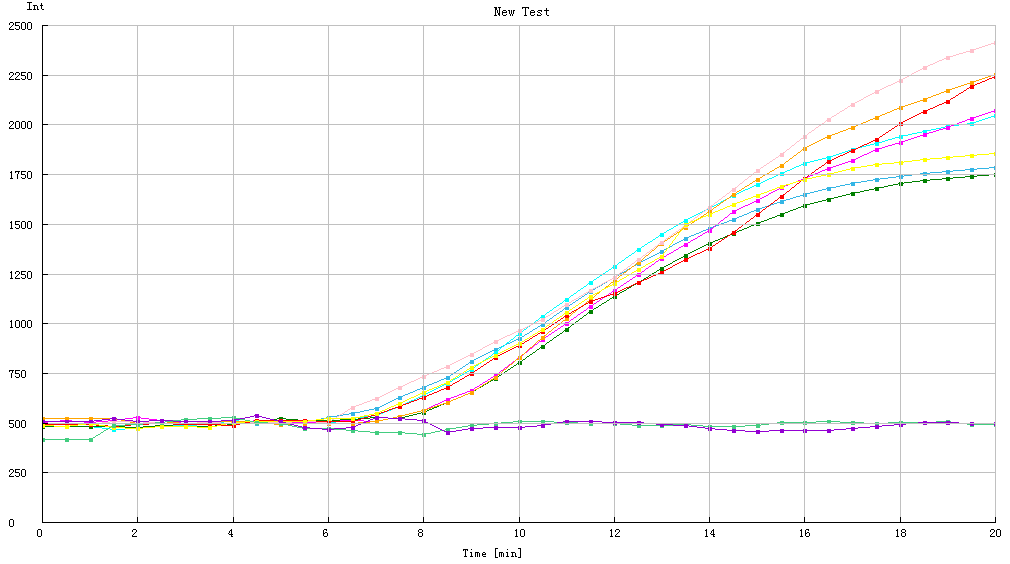


Fig S3 Eight replicates of RNA standard (10^4^ copies/μL) were detected by the assay.

Fig S4


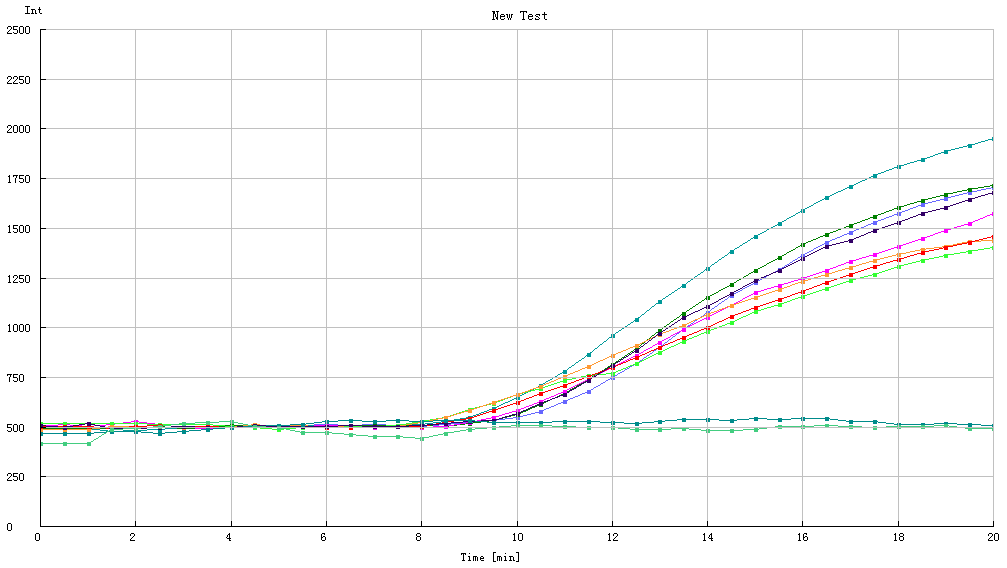


Fig S4 Eight replicates of RNA standard (10^3^ copies/μL) were detected by the assay.

Fig S5


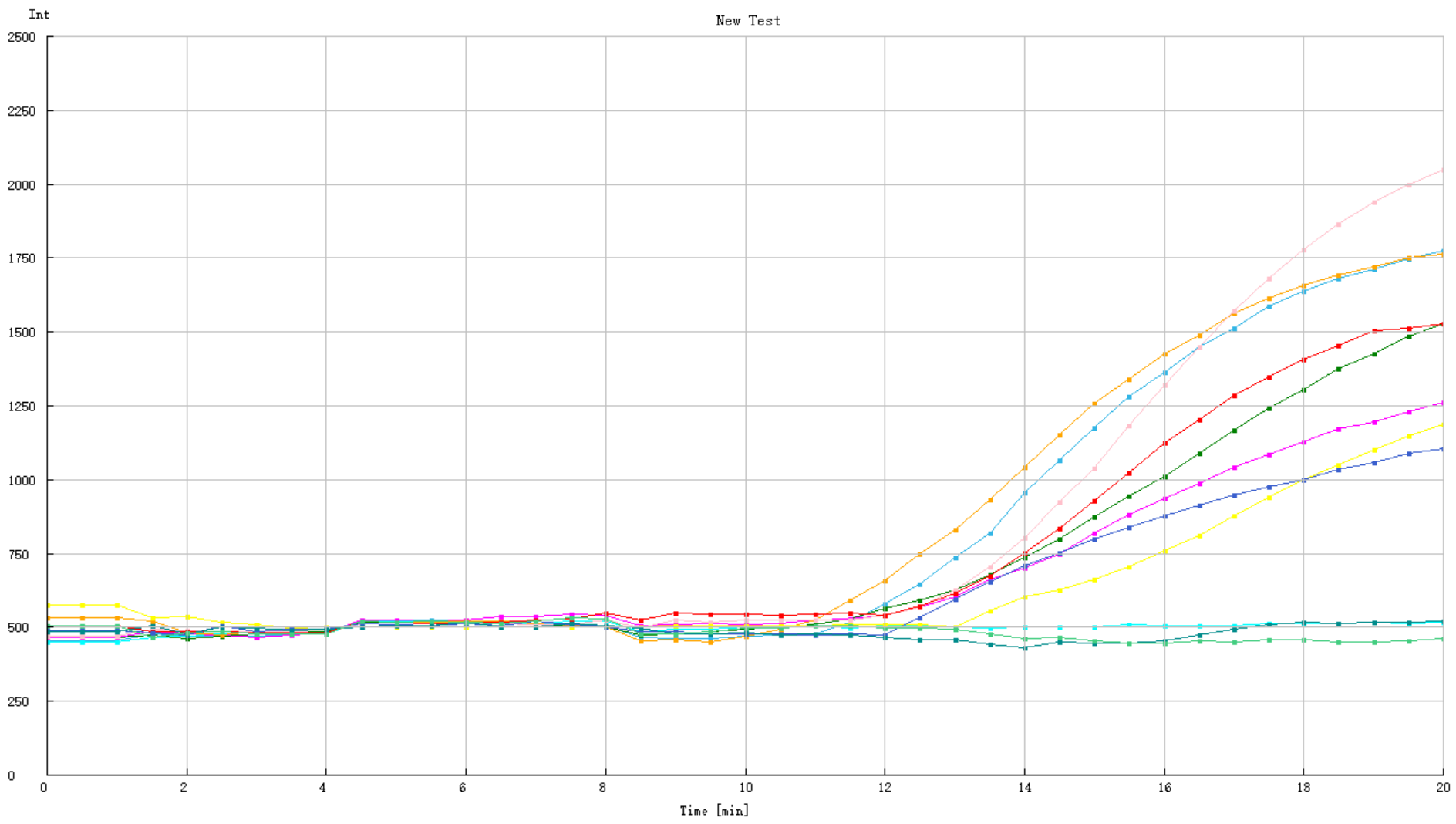


Fig S5 Eight replicates of RNA standard (10^2^ copies/μL) were detected by the assay.

Fig S6


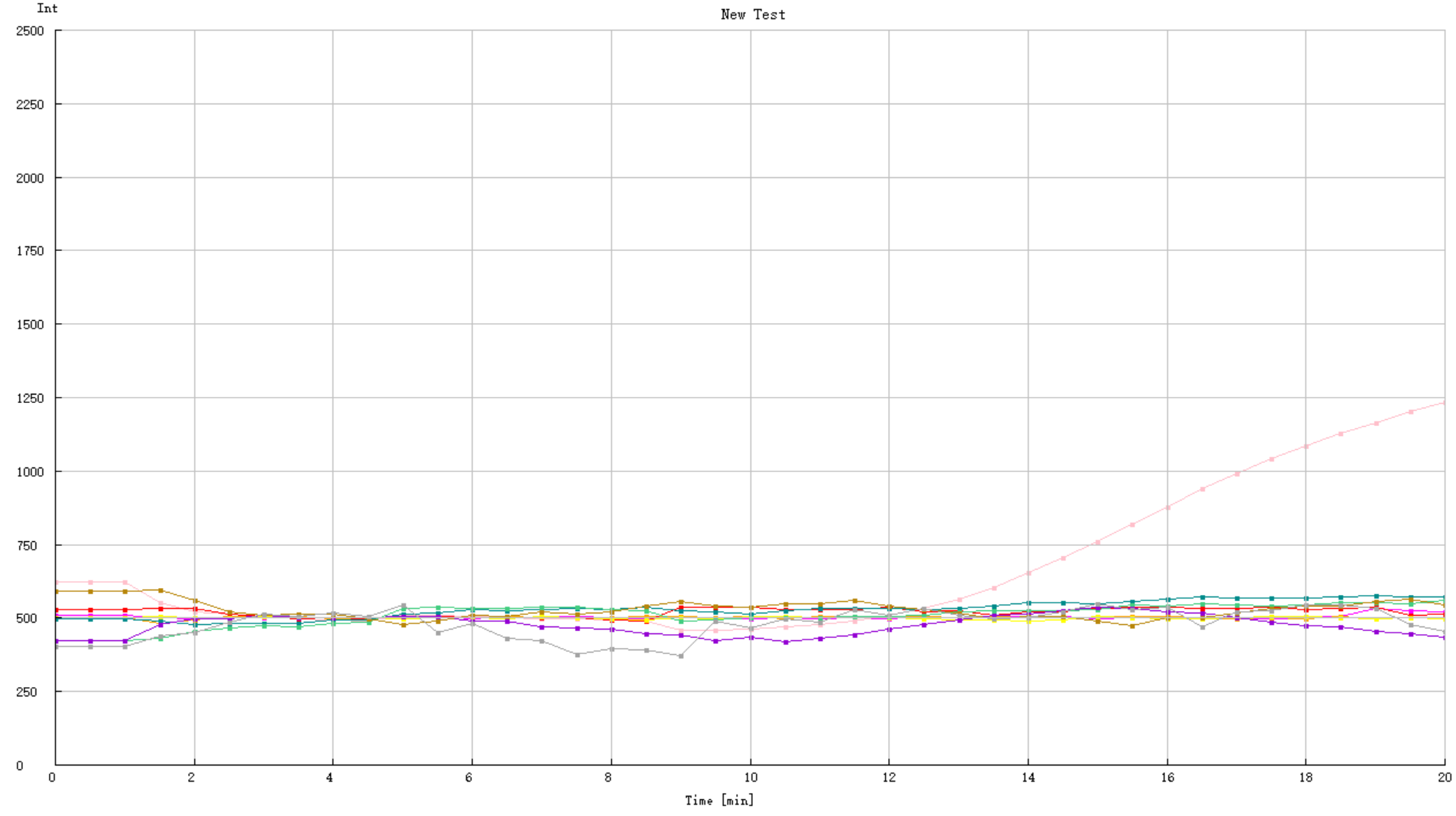


Fig S6 Eight replicates of RNA standard (10 copies/μL) were detected by the assay.

Fig S7


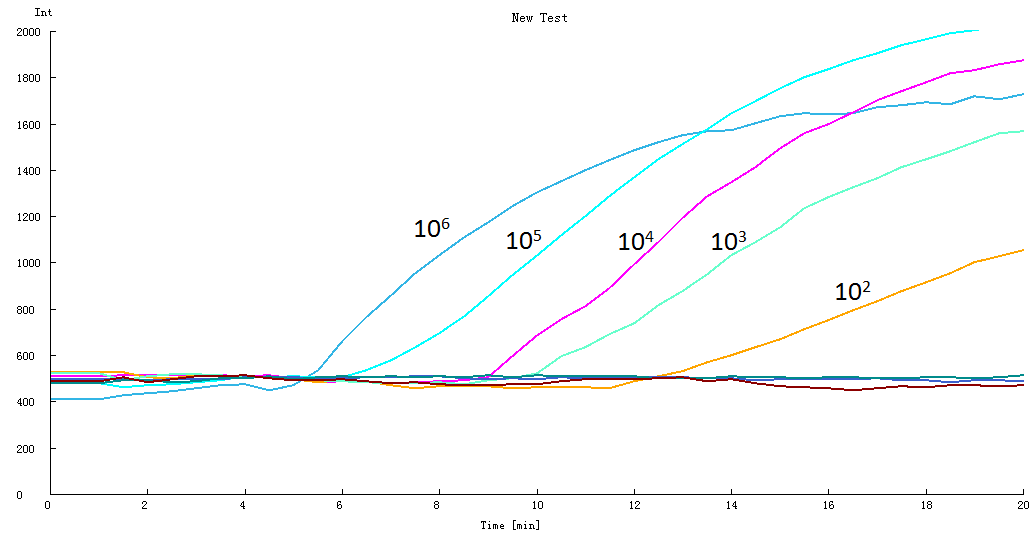


Fig S7 The sensitivity of the assay using ARV strain GX110058 segment S1 as the template.

Fig S8


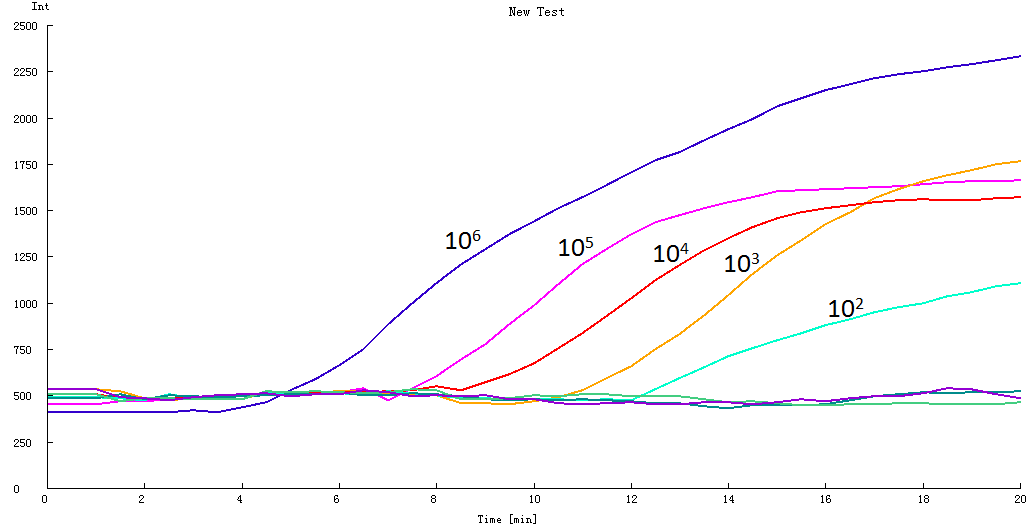


Fig S8 The sensitivity of the assay using ARV strain strain B-98 segment S1 as the template.
